# Textural, Rheological and Sensory Properties and Oxidative Stability of Nut Spreads—A Review

**DOI:** 10.3390/ijms14024223

**Published:** 2013-02-20

**Authors:** Ahmad Shakerardekani, Roselina Karim, Hasanah Mohd Ghazali, Nyuk Ling Chin

**Affiliations:** 1 Iranian Pistachio Research Institute, Rafsanjan, Kerman 77175-435, Iran; E-Mail: shaker@pri.ir; 2 Department of Food Technology, Faculty of Food Science and Technology, University Putra Malaysia, Selangor 43400, Malaysia; 3 Department of Food Science, Faculty of Food Science and Technology, University Putra Malaysia, Selangor 43400, Malaysia; E-Mail: hasanah@putra.upm.edu.my; 4 Department of Process and Food Engineering, Faculty of Engineering, University Putra Malaysia, Selangor 43400, Malaysia; E-Mail: chinnl@eng.upm.edu.my

**Keywords:** roasting, milling, stability, colloid, quality, rancidity, peanut butter, rheology

## Abstract

Tree nuts are rich in macro and micronutrients, phytochemicals, tocopherols and phenolic compounds. The development of nut spreads would potentially increase the food uses of nuts and introduce consumers with a healthier, non-animal breakfast snack food. Nut spreads are spreadable products made from nuts that are ground into paste. Roasting and milling (particle size reduction) are two important stages for the production of nut spreads that affected the textural, rheological characteristic and overall quality of the nut spread. Textural, color, and flavor properties of nut spreads play a major role in consumer appeal, buying decisions and eventual consumption. Stability of nut spreads is influenced by its particle size. Proper combination of ingredients (nut paste, sweetener, vegetable oil and protein sources) is also required to ensure a stable nut spread product is produced. Most of the nut spreads behaved like a non-Newtonian pseudo-plastic fluid under yield stress which help the producers how to start pumping and stirring of the nut spreads. Similar to other high oil content products, nut spreads are susceptible to autoxidation. Their oxidation can be controlled by application of antioxidants, using processing techniques that minimize tocopherol and other natural antioxidant losses.

## 1. Introduction

In general, tree nuts are dry fruits with one seed in which the outer wall becomes hard upon maturing. The most popular tree nuts in the world are almond, Brazil nut, cashew, hazelnut, macadamia, pecan, pine nut, pistachio and walnut. Considering the production of world’s most popular tree nuts, cashew nut ranks first on a global basis with a production of 2,760,000 MT, followed by almond (2,560,000 MT), walnut (2,550,000 MT), Brazil nut (1,000,000 MT), pistachio (940,000 MT) and hazelnut (860,000 MT) in 2010. The production of remaining tree nuts (pecan, macadamia, and pine nut) was around 1.85 MT in the same year [1]. In addition to the main composition (Table 1), some phytochemicals such as tocopherols and phenolic compounds are present in tree nuts [2–6]. Although peanut is a legume, it shares a similar nutrient profile with tree nuts. Therefore, it is used as comparison with tree nuts for the purpose of this review. With today’s busy lifestyles, tree nuts are nutritious, tasty, convenient, and easy snack that contribute to a healthy lifestyle. They are typically consumed as whole nuts (either raw or roasted or salted) or used as ingredients in a variety of processed foods, especially in spreads, bakery, and confectionary products, among others. Tree nut oils are also used for several purposes such as cooking, salad dressings, and flavoring ingredients, among others. In addition, tree nut oils are also components of some skin moisturizers and cosmetic products [2]. According to FAO (2012), the average nut consumption in the world is 2.1 kg/person per year [1]. Tree nut consumption varies both among and within the regions where there is tree nut production. For instance, the European region is shown as having consumption of 2.8 kg/person per year. However, when looking more closely at individual country consumption within that region, Spain and Greece (tree nut producers) have consumption levels of 7.3 and 9.9 kg/person, respectively. The variations in consumption between North and South Europe are even more apparent in the food availability (as kg/cap/year) with EU (4.0), Spain (6.7) and Greece (10.0). Tree nuts are five times more available to Greeks than U.S. consumers. Consumption levels in Asia, with vast population levels, are even more skewed [7].

The consumption of nuts in roasted and salted form is limited. Children and elderly people cannot easily open the nuts (such as pistachio) and consume them. Another problem after processing and before consumption of nuts is their storage and handling which influence the quality of the product. There are many reports about presence of mycotoxins, especially aflatoxin in nuts [9–11]. Increases in the moisture content, air temperature, and air relative humidity are the main reasons for increasing the fungal growth and as a result of aflatoxin production [12]. There is some evidence that contamination occurs during the export process, the sea transport or storage at an imported countries [13]. If the total mycotoxins (especially aflatoxin B_1_) level of nuts increased to more than the maximum allowance level, the nuts cannot used by the consumers. There are some reports of rejection of exported nuts due to aflatoxin levels [13,14]. Development of new products (such as nut spread) from nuts and using suitable packaging materials can reduce the risk of losses of product due to contamination to mycotoxins. Development of nut spread would potentially increase the food uses of nuts and introduce consumers to a healthier, non-animal breakfast snack food. In this review, the production of the stable nut spreads and factors affecting their quality will be explained. The effect of processing conditions (roasting and milling) on the rheological behavior, sensory acceptance and oxidative stability of nut spread were also presented in this review.

## 2. Nut Butters and Nut Spreads

The term “nut butter” refers to a product that contains at least 90% nut ingredients whereas, the term “nut spread” refers to a spreadable product having at least 40% nut ingredients, which can be added in various forms, e.g., as nuts, a paste and/or a slurry [15,16]. Nut butters and nut spreads are spreadable products made from nuts that are ground into paste. Both nut products can be spread like commercially available butter. They can be produced from almond, cashew, hazelnut, macadamia nut, peanut, pecan, pistachio and walnut. Similar spreads can also be made from other seeds such as sesame seed, pumpkin seed, soybean and sunflower seeds, but they are not categorized as nut spread [17]. Nut spreads have a variety of uses, and the most common use of nut spreads is in sandwich preparation. Other uses include as toppings for edible crackers or as dips for vegetable pieces. Besides that, nut spread is also used in a variety of baking and cooking applications. Nut spread is popular and widely accepted by consumers due to its flavor, good nutritional values and suitability for consumption either alone or in combination with a variety of other foods. The nut-based spreads market in the US increased at a compound annual growth rate of 6.2% between 2004 and 2009 [18]. Since the most important characteristic of nut spread is spreadability, it is of utmost importance that the product should have a soft texture and be easily spreadable to avoid tearing the bread or crumbling the crackers. In addition, since children are the most popular user of nut spread, soft and spreadable product characteristics will help to facilitate the application of nut spread by this age group without assistance from their parents. For this reason, creamy and smooth nut spreads are preferred.

## 3. Nut Spread Production

In general, the quality of nut spreads depends on the formulations used (Table 2).

There is a wide variation in the quality of nut spreads as many types of ingredients can be used for the production of spread. Proper combination of these ingredients during production is required to ensure a stable nut spread product. The basic formulation of nut spread usually contains the following ingredients: (a) selected, blanched, dry roasted nuts; (b) sweeteners; (c) vegetable oils; (d) emulsifiers; (e) protein sources; and (f) flavorings [23,25].

One of the famous nut spreads is prepared from peanuts. According to the USDA, peanut butter and peanut spread shall conform to the classification shown in Table 3.

**Figure 1:**
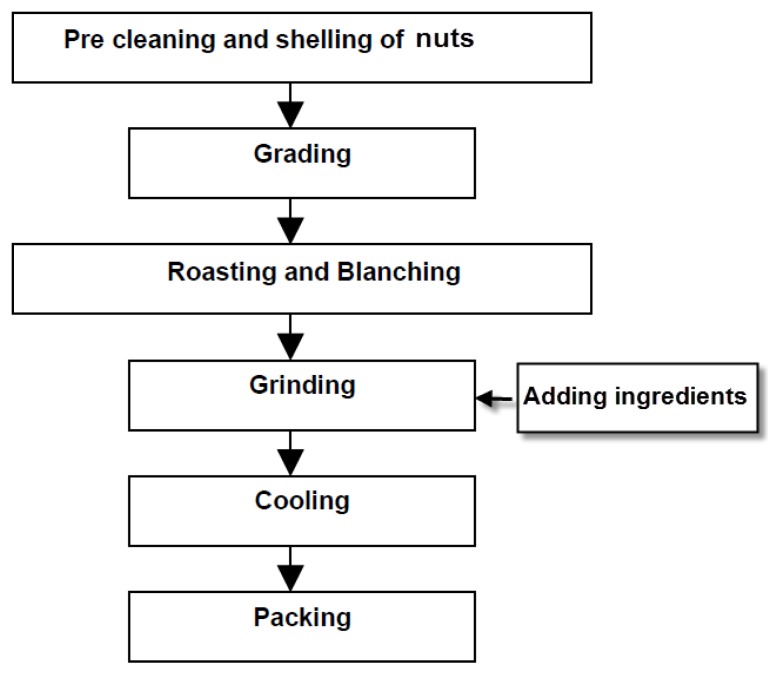
Flow diagram of nut spread production.

Peanut spread is a paste made from ground roasted peanuts, with or without addition of oil. It is popular across the world and is produced in some emerging markets. It is used as a sandwich spread. Besides peanuts, other types of nuts can be used to make nut spreads [26–28]. Nut spreads are sometimes presented with layers of either jam or jelly in a bottle [29]. These types of layered spread are normally consumed with bread as sandwiches. Figure 1 shows a simple flowchart of nut spread production.

Table 4 summarizes the steps involved in nut spread production, their functions and characteristics of process. Roasting and milling (grinding) are two important stages in nut spread productions.

### 3.1. Roasting of Nuts

Raw nuts contain lipoxygenase, which accelerate the oxidation of damaged nuts [22]. The lipoxygenase is usually destroyed during nut roasting, but after roasting, non-enzymatic catalysts can initiate oxidation [23]. The high unsaturated fatty acid content of nut spread makes it sensitive to oxidation [24]. Metalloproteins together with copper and iron salts are major catalysts in oxidation of fatty acid in nut spread [25]. The roasting condition for nut spreads depends on the type of nut and type of roaster. Birch *et al.* (2010) pointed out that the most suitable roasting temperature and time for macadamia nuts were 135 °C and 20 min, respectively [28].

In contrast, the best roasting conditions for peanuts were at 180 °C for 45 min [26] or 160 °C for 40–50 min [27]. The textural characteristic of the nut is affected by the roasting condition. During the roasting process, the moisture content of nuts was reduced [34] and the texture became more fragile and crumbly [35]. Hardness property was used as an indicator of textural quality in peanuts [36]; and pistachio nuts [37,38] during roasting. The roasting condition of kernels should be properly controlled because it affected the development of flavor, aroma and also the color of the final pistachio paste. Color is a significant quality indicator of the roasting process. During roasting, browning reactions and caramelization occur and brown pigments are formed [39]. The effect of roasting conditions on color changes were reported by several researchers in their studies on hazelnuts [40,41], peanuts [42], sesame seeds [43] and macadamias [44]. Roasting conditions also influenced the storage stability of nuts [37,38].

### 3.2. Milling of Roasted Nuts

Milling process is carried out to reduce the size of kernel for the production of pistachio paste. This step is critical in the production of nut spread because the particle size and particle distribution are important parameters that influence the overall quality of final product [45]. Optimizing the distribution of particle size in suspensions resulted in 50 fold reductions in the shear viscosity [46]. The particle size distribution and rheological behavior of semi solid food products determine their preparation, processing and storage stability [47]. The influence of particle size distribution on the stability and rheological properties of food colloids such as chocolate, peanut butter and sesame paste had been reported by Dickinson (2010), and Genovese *et al.* (2007) [48,49]. Ciftci *et al.* (2008) observed that reduction in particle size of sesame paste increased the stability of the product [50]. Another study had shown that if an almond paste contained a substantial number of particles having a diameter of over 105 μm, the coarser particles will precipitate indicating the instability of the paste [51]. According to Wong *et al.*, in order to reduce the grittiness of peanut butters, the average particle size must be about 20 μm [52]. Wong *et al.* (1992) employed a series of colloid mills or homogenizer to obtain the desirable particle size and viscosity of peanut butters [21]. According to Liedl and Rowe (2007) an acceptable sensory and textural characteristics of nut spread can be produced if the particle size distribution of the product was as follows: at least 90% of the particles were smaller than 40 μm, 50% smaller than 10 μm and 10% smaller than 3 μm [23].

## 4. Quality of Nut Spreads

The overall quality of nut spread is related to the quality of nut paste which is used as the main ingredient. The quality of nut paste is influenced by raw kernel quality, processing conditions such as roasting temperature and time, and storage conditions [29]. Efforts have been made to improve the flavor and texture of nut spreads by addition of moist ingredients such as honey or several types of flavorings. In product development, roasted nut may be used in high moisture systems such as commercial nut spread and jelly. It had also been reported that the shelf life of nut spread depends on the type of ingredients added to the nut paste during production of the nut spread [53,54]. Table 5 lists the quality parameters or attributes of different types of spreads found in the literature.

## 5. Rheological Properties of Nut Spreads

Rheology is the science of flow and deformation of materials under stress and strain. In the food industry, rheological data are needed for studying the functionality of ingredients in product development, determination of food texture by correlation to sensory data, immediate or final product control and process engineering calculation for equipment such as pumps, heat exchangers, extruders and mixers. Many semi solid food products are made of dispersions of colloidal sized particles such as solids or immiscible liquids and their polymers. The presence of these suspended particles and polymers may affect the stability and the rheology of the suspensions as a result of interactions between them. Several works on the rheological properties of semi solid pastes such as peanut butter [45] and pistachio butter [68,69] had been reported. Citerne (2001) reported that peanut butter behaved like plastic material and apparent yield stress of 24 Pa and 370 Pa for the unstablized and the stabilized suspensions, respectively [45]. Taghizadeh and Razavi (2009), in their study on the time independent rheological properties, reported that pistachio butter behaves like a non-Newtonian pseudo-plastic fluid under yield stress [68]. The yield stress can be used to calculate whether a sample is likely to settle *in situ*, or whether it will be difficult to start pumping or stirring. Good rheological product design will enhance processing and end use. Although many researchers have investigated the time-dependent rheological behavior of materials, in general, the thixotropic characteristics of many other foodstuffs in food processing are not extensively studied. Viscoelasticity is also one of the rheological properties of materials. In viscoelastic tests, the elasticity part is defined as storage modulus (G’), the viscosity part as loss modulus (G”) and ratio of G”/G’ as damping factor. Shakerardekani (2012) reported that storage modulus, loss modulus and damping factor for pistachio paste were 42943 Pa, 6370 Pa and 0.15, respectively [70]. According to Rao (2007), in any flow process, whether during manufacturing and masticating of the food product, the flow stress affects the structure of the system, which, in turn, affects its rheological characteristics [71]. A rheological analysis of structured water-in-oil emulsions consist of a network of solid fat particles in a continuous oil phase with water droplets captured in it. Chocolate is an example of fat continuous food dispersion, where a continuous network is formed by solid fat, sugar, protein particles and ground cacao particles. All of these systems contain emulsifiers as stabilizers and rheology regulators or fat crystallization regulators. The emulsifiers are effective mainly because of their ability to absorb to different interfaces [72]. All emulsion exhibited a gel-like characteristic with storage modulus higher than loss modulus. Understanding the rheology of emulsions is crucial in predicting the long-term instability of the product particularly due to flocculation and coalescence. Rapid increase in the storage modulus upon ageing can be an indication of strong flocculation [73]. The flocculation of oil droplets usually resulted in liquid entrapment and increases the effective volume fraction of the emulsion. Hence, the increase in the net attraction among droplets resulted in the increase in storage modulus. In contrast, ageing of the emulsions decreases the storage modulus due to droplet rearrangements that led to a weaker structure [74,75].

## 6. Sensory Evaluation of Nut Spreads

A broad spectrum of sensory characteristics, including appearance, aroma, flavor, and texture are used by consumers to make purchasing and consumption decisions related to foods [76]. Trained panelists describe a product’s behavior in their mouth in terms of quality and quantity through its mechanical, geometrical, and fat and moisture characteristics from the first bite through complete mastication. Several studies had reported on the usage of Quantitative descriptive analysis (QDA) for characterization of the sensory properties of peanut butter, peanut spread and peanut soy spread [55,66,77,78]. According to McNeill *et al.* (2002) among important qualities of peanut butter include texture, color, flavor and nutritive value [66]. Texture is one of the sensory properties of foods that play a major role in consumer appeal, buying decisions and eventual consumption. It was found to be the single most dominant attribute of consumer preference of foods [79]. Spreadability is an extremely important attribute of semi-solid food texture. Spreadability is a subjective term related to how easy a sample is uniformly distributed over a surface. Gills & Resurreccion reported that descriptive attributes spreadability highly correlated with consumer attribute spreadability [77]. Beside textural attributes, overall liking of spreads such as hazelnut spread is related to the flavor of the product [27]. Flavor liking cannot be measured directly by instruments; it is an interaction of consumer and product [80]. With a wide range of materials and processing methods in peanut butter manufacturing, processors may not have a clear understanding of the most desirable roasting or storage conditions that contribute to the flavor of their product [81,82]. Pattee *et al*. (1991) [83] showed that the peanut flavor (nutty, sweet, salty, roasted and rancidity) from sensory analysis is related to the roasted peanut. In the case of roasted nuts, the volatile profiles are highly complex and are composed of compounds arising not only from lipid oxidation, but also from Maillard reaction, Strecker degradation, and caramelization of sugars [84]. Color is another important attributes used by consumers to judge the acceptability of food products [85]. It can be concluded that sensory evaluation can be used to provide further information on the texture, color, flavor and as a result acceptability of nut spread by consumers.

## 7. Oxidative Stability of Nut Spreads

Lipid oxidation is initiated by compounds known as sensitizers which include heat, light and metal ions. Lipid oxidation produces undesirable flavors, aromas and compromises the nutritional quality of fats and oils leading to the production of toxic compounds [86]. The changes in fatty acid composition of lipids provide an indirect measure of susceptibility to lipid oxidation. Nuts may be held for up to 2.5 years under optimum conditions, but under unsuitable storage conditions they become inedible within a month either due to insects, mold, absorption of foreign flavors, discoloration, staleness or rancidity. Fat content alone is not a good indicator of storage stability [87] but the degree of unsaturation or polyunsaturation [88,89], tocopherols [87], chlorophyll and beta carotene, moisture content [90] and temperature [91,92] affect primary lipid oxidation and oxidative stability of intermediate moisture foods during storage. In the presence of oxygen, oxidative reactions are usually of the greatest importance and hence, the storage life is then limited by the development of oxidative rancidity of fat in the food product [93,94].

The protective effects of various antioxidants on the oxidative stability of plant-derived oils and fats have been the subject of intense research, showing great potential to protect oils and fats against oxidation. The use of natural antioxidants and natural products has been widely studied by several researchers [95–97]. Judde *et al.* (2003) found that the addition of 1% (*w*/*w*) soy lecithin was effective in delaying the oxidation in rapeseed, soy, walnut, and palm oils by increasing the induction time by 1.7–1.8 times when measured at 110 °C and decreasing the peroxide values by 2.2–4.6 folds when heated at 40 °C for 35 days [98]. According to Miraliakbari & Shahidi (2008), the oils of pecans and pistachios were the most stable, whereas oils of pine nuts and walnuts were the least stable due to unsaturated fatty acid level [99]. The oxidation of oils and fats can be controlled by application of antioxidants, using processing techniques that minimize tocopherol and other natural antioxidant losses [86,100,101].

## 8. Strengths, Weakness, Opportunities and Threats of Nut Spread Industries

Table 6 shows strengths, weakness, opportunities and threats of nut spread industries. Some Strategies for maximizing strengths and opportunities and mitigating weaknesses and threats of nut spread industry are listed in Table 7.

## 9. Gaps in Nut Spread Production

The main gaps in nut spread production are as follows:

### 9.1. Oil Separation from the Nut Spreads

Since most nut spreads are rich in oil, oil separation is one of the problems faced by this industry. The separated oil contaminates the packaging material and affected the quality and appearance of nut spreads [105]. Stabilizing against oil separation is of utmost important to improve the acceptability and marketability of these products [106]. Although the spread manufacturers have been using different commercially available food additives or their combinations to solve the oil separation problem, it was observed that this problem had not been completely overcome. In addition, it was also determined that some of these additives were not permitted to be used in these products. Reports on oil separation problem in spreads are very limited. Ereifej *et al.* (2005) investigated the effects of various additives such as palm oil, soy protein isolate, gelatin, lecithin, gum arabic, pectin, and ground sugar on oil separation in tahin helva (sesame spread) [105]. They found that sugar powder, gum arabic and pectin decreased oil leakage; however, this decrease was not significant compared to the control. Nut spreads may contain an emulsifier at a level of 0.1% to 3% of the total ingredient. Emulsifiers help to prevent and improve the overall texture of the product. Most of the commercial emulsifiers are distilled monoglycerides, and contain a minimum of 90% monoglycerides. In selecting a suitable emulsifier, the organoleptic properties must be considered. Monoglycerides are usually preferred but lecithin in combination with monoglycerides may also be used in the production of nut spreads [107,108].

### 9.2. Allergy to Nut Spreads

Prevalence of food allergy in the United States is about 6% in children (≤5 years) and 3.7% in adults [109]. More than 75% of total food allergies in young children are attributed to milk (41%), egg (21%), and peanuts (13%), whereas shellfish (54%), peanuts (16%), and tree nuts (13%) account for about 85% of the total food allergies in adults [110]. While tree nuts have been shown to play a protective role is health and disease, it is important to note that they can also induce adverse reactions in susceptible individuals. Tree nuts are one of the most allergy-causing foods with symptoms varying substantially depending on the individual, ranging from hives, itching, and swelling to life-threatening anaphylaxis. It is estimated that 12 million Americans have food allergies with 3.3 million allergic to peanuts or tree nuts (1.1% of the population) [2]. Every year in the United States, food allergies result in over 30,000 emergency room visits and between 150 and 200 fatalities. Of the Americans with peanut or tree nut allergies, 50% were reactive to peanuts, 30% to walnuts, 10% to almonds, and 4% to both peanuts and tree nuts and 10% of allergic individuals were reactive to two or more nuts [2]. Lack of proven medical treatments for food allergies dictates that avoidance of the offending food to be the best choice for sensitive individuals.

### 9.3. Aflatoxin Contamination of Nut Spreads

Aflatoxin is one of the serious constraints in the marketing of tree nuts. Contamination of human foods and animal feeds by these compounds has become an important international food safety and trade issue since aflatoxins are considered to be potent carcinogens to humans [11,111]. Research has also shown that aflatoxin production is markedly decreased by the presence of natural antioxidants that occur in tree nuts, including hydrolyzable tannins, flavonoids, and phenolic acids [112]. Application of good manufacturing practice (GMP), goof hygiene practice (GHP), good storage practice (GSP) and hazard analysis critical control point (HACCP) can prevent aflatoxin contamination of tree nuts.

### 9.4. Salmonella Contamination of Nut Spreads

The ability of salmonella to survive in high fat content, low water activity foods like peanut butter has been demonstrated by large foodborne illness outbreaks in recent years [113]. About 1.2 billion pounds of peanut butter are consumed annually in the United States. In 2008 to 2009, an outbreak involving *Salmonella typhimurium* in peanut butter led to a recall of over 3900 products by over 200 companies. More than 700 people became sick, 100 were hospitalized, and 9 people died from this outbreak [114]. Using Thermal treatments [115], high-pressure processing (HPP) [113,114] and production of nut spreads with lower moisture content [116] can reduce the risk of salmonella contamination in nut spreads specially peanut spread.

## 10. Potential Studies of Nut Spreads in the Future

Recently, much consumer attention has focused on those who follow reduced carbohydrates and fats or protein and vitamin fortified nut spreads. Low-calorie pistachio butter [117], milk-based fortified spreads [118], peanut spreads fortified with soy flour [119] and protein fortified peanut butter [120] are examples of these nut spreads. While nut spreads tend to be relatively low in carbohydrates, in view of the recent focus by many on minimization of carbohydrate intake, it would be desirable to reduce further the level of carbohydrates in such products. Unfortunately, consumers show little liking for losing organoleptic properties in their favorite foods. Therefore, low carbohydrate products should have good organoleptic properties similar to their full carbohydrate nut spreads. Much of the peanut butter literature focuses on achieving good spreadability and mouth feel, avoiding oil separation, and reducing fat or total calorie content. Particularly in view of the recent attention paid to carbohydrate and fat reduction, there is still a need for nut spreads which are neatly and easily applied and which have reduced levels of fats and carbohydrates.

## 11. Conclusions

Nut spreads are spreadable products made from roasted nuts. In addition to the ingredients (nuts, sugar, vegetable oil, and emulsifier), the roasting conditions of nuts, particle size distribution and type and amount of stabilizer affected consumers acceptability, rheological behavior and oxidative stability of nut spreads. The oxidation of oils and fats can be controlled by application of antioxidants, using processing techniques that minimize tocopherol and other natural antioxidant losses. Recently, much consumer attention has focused on reduced carbohydrate and fat nut spreads. Although much of the literature has reported on nut spread production, most studies were related to peanut butter and peanut spread. It is recommended that further research be undertaken to develop other types of nut spreads with prolonged shelf life.

## Figures and Tables

**Table 1:** Nutritional composition of tree nuts and peanuts (per 100 g) [8].

Nutrient	Almond	Brazil nut	Cashew	Hazelnut	Macadamia	Peanut	Pecan	Pine nut	Pistachio	Walnut
Calories (kcal)	578	656	574	628	718	567	691	629	557	654
Protein (g)	21	14	15	15	8	26	9	12	21	15
Total fat (g)	51	66	46	61	76	49	72	61	44	65
Saturated (g)	4	16	9	4	12	7	6	9	5	6
Monounsaturated (g)	32	23	27	46	59	24	41	23	23	9
Polyunsaturated (g)	12	24	8	8	2	16	22	26	13	47
Carbohydrate (g)	20	13	33	17	14	16	14	19	28	14
Dietary fiber (g)	12	5	3	10	9	9	10	11	10	7

**Table 2:** Type of ingredients (%) for production of nut spreads and nut butters.

Nut	Oil	Stabilizer	Sweetener (Sugar)	Salt	Emulsifier	Soy protein	Reference
79.0	13.1	-	6.0	1.4	0.5	-	[19]
83.6	6.5	2.1	6.8	0.9	0.2	-	[20]
73.8	17.2	-	6.3	0.9	0.2	-	[21]
71.6	10.3	2.1	4.2	1.0	-	-	[22]
59.2	2.1	0.9	32.5 **	-	-	5.3	[23]
86.3	5.0	1.0	6.2	1.5	-	-	[24]

** include 25.3% maltodextrin and 7.2% sugar.

**Table 3:** Classification of peanut butter and peanut spread [16].

Style	Peanut butter	Peanut spread
**Class A**	**Regular**	**Regular**
Texture 1	Smooth	Smooth
Texture 2	Medium	-
Texture 3	Chunky/crunchy	-
(i) Type a	Stabilized	Stabilized
Flavor 1	-	Plain
Flavor 2	-	Chocolate
Flavor 3	-	Other
(ii) Type b	Non-stabilized	-
Fortification a	Non-fortified	Non-fortified
Fortification b	Fortified	Fortified
**Class B**	**Reduced Fat**	**Reduced Fat**
Texture 1	Smooth	Smooth
Texture 2	-	Chunky/crunchy
(i) Type a	-	Stabilized
(ii) Type b	Non-stabilized	-
Fortification a	Non-fortified	Non-fortified
Fortification b	Fortified	Fortified

**Table 4:** Stages of nut spread production [25,30–33].

Stage	Function	Explanation
1. Roasting	To reduce moisture content and develop flavor	For peanuts, 160 °C for 40–50 min is required depending upon the initial moisture contents
2. Blanching	To separate hulls	In most nuts, a white colored nut will be obtained
3. Picking and inspection	To remove damaged nuts and foreign matter	To obtain good quality raw material
4. Grinding	To form a fine and smooth texture	Sugar or other sweeteners is usually added at this stage (optional)
5. Adding ingredients	To produce final product	Adding of the remaining ingredients to the heated slurry prior to mixing
6. De-aeration	To remove air	Removal of air using a vacuum kettle
7. Cooling	To prepare a stable product	Carried out using scraped surface heat exchanger
8. Filling and packing	To prepare for dispatching	The product is allowed to set at 20 °C for ~35–40 h before distribution

**Table 5:** Quality parameters of different types of nut spreads and butters.

Type of product	Parameters/Attributes of product	Reference
Peanut soy spread	Hardness, cohesiveness, adhesiveness, gumminess and aroma	[55]
Peanut butter	Water activity, color, hardness	[56]
Peanut butter	Particle size	[57]
Peanut butter	Particle size, salt and sucrose concentrations	[58]
Peanut butter	Oiliness, firmness, cohesiveness, adhesiveness, stickiness	[59]
Peanut butter	Hardness, oiliness, spreadability, brown color	[60]
Peanut butter	Oil content, particle size	[45]
Peanut butter	Oil separation	[61]
Peanut paste	Moisture content, sugar content	[54]
Peanut-sesame-soy spread	Sensory attributes (roast peanut, sweetness, bitterness)	[62]
Peanut spread	Peanuty, buttery, oxidized, sweet, salty, sour, bitter	[63]
Peanut butter tart	Color, consumer acceptability (appearance, flavor, texture)	[64]
Peanut butter	Water activity	[29]
Hazelnut butter	Peroxide value, sensory test (color, flavor, taste)	[65]
Nut spread	Roasting attributes (brown color, roasted taste, burnt taste)	[32]
Peanut butter	Appearance, aroma, flavor	[66]
Pistachio butter	Oil separation	[28]
Pistachio butter	Viscous flow behavior	[67]
Pistachio butter	Level of emulsifier, rheological model	[68]

**Table 6:** Strengths, weakness, opportunities and threats of nut spread industries [102,103].

**Strengths**	Tasty, nutritious and healthy. Products perceived as the economical choice. Strong image as socially responsible. Significant resources through grants. Complementary product mix. Good client relationships. Good source of protein for athletes.
**Weaknesses**	Limited advertising and penetration in emerging economies. Apprehension about its health effects leading to loss of trust. Oil separates from peanut butter. Quality is inconsistent. Lack key management and technical expertise. Difficult to achieve economies of scale.
**Opportunities**	Different flavors *i.e*., combination of nuts and chocolate. Increase in disposable income in developing countries. Readiness and attraction to adapt to western styles of breakfast. Growing demand for processed foods. Locally available raw materials. Most similar products are imported. Rising consciousness of social responsibility at retailer level.
**Threats**	1. Change in perception of the consumers around the world following the recent lawsuit. 2. Some families may not like chocolate for breakfast. 4. Allergies, especially to peanut spread. 5. Salmonella scare specially for peanut spread. 6. Aflatoxin scare. 7. People eating other snacks such as Nutella. 8. Lack of advertising/innovation.

**Table 7:** Strategies for maximizing strengths and opportunities and mitigating weaknesses and threats of nut spread industry [102,104].

Aim	Strategies
Maximizing strengths and opportunities	Leverage nut spreads’ social contribution to secure contracts with socially conscious retailers who are familiar with nut spreads products and get other retailers to try carrying products on this premise. Plow resources into marketing efforts to get consumers to switch to nut spreads and increase its brand recognition across markets. Nut spread will position itself as the economic choice and compete on price against other similar products. Secure contracts with producers guaranteeing their market and increasing supply of raw materials.
Mitigating weaknesses and threats	Hire needed staff in production and marketing from private industry. Invest in product development to improve product quality and meet market demand. Examine possibilities to increase economies of scale by investing in equipment or electrifying production center and adding evening shifts. Contract technical assistance from specialists to alleviate technical knowledge gaps; specifically to deal with problem of oil separating. Hire guard to improve security situation. Application of good manufacturing practice (GMP), goof hygiene practice (GHP), good storage practice (GSP) and hazard analysis critical control point (HACCP) to prevent aflatoxin and salmonella contamination.
